# Amyloid-β Receptors: The Good, the Bad, and the Prion Protein*[Fn FN2]

**DOI:** 10.1074/jbc.R115.702704

**Published:** 2015-12-30

**Authors:** Heledd H. Jarosz-Griffiths, Elizabeth Noble, Jo V. Rushworth, Nigel M. Hooper

**Affiliations:** From the ‡ Institute of Brain, Behaviour and Mental Health, Faculty of Medical and Human Sciences, University of Manchester, Manchester M13 9PT and; the § Faculty of Health and Life Sciences, De Montfort University, Leicester LE1 9BH, United Kingdom

**Keywords:** Alzheimer disease, amyloid, oligomer, prion, receptor

## Abstract

Several different receptor proteins have been identified that bind monomeric, oligomeric, or fibrillar forms of amyloid-β (Aβ). “Good” receptors internalize Aβ or promote its transcytosis out of the brain, whereas “bad” receptors bind oligomeric forms of Aβ that are largely responsible for the synapticloss, memory impairments, and neurotoxicity that underlie Alzheimer disease. The prion protein both removes Aβ from the brain and transduces the toxic actions of Aβ. The clustering of distinct receptors in cell surface signaling platforms likely underlies the actions of distinct oligomeric species of Aβ. These Aβ receptor-signaling platforms provide opportunities for therapeutic intervention in Alzheimer disease.

## Introduction

**FIGURE 1.**
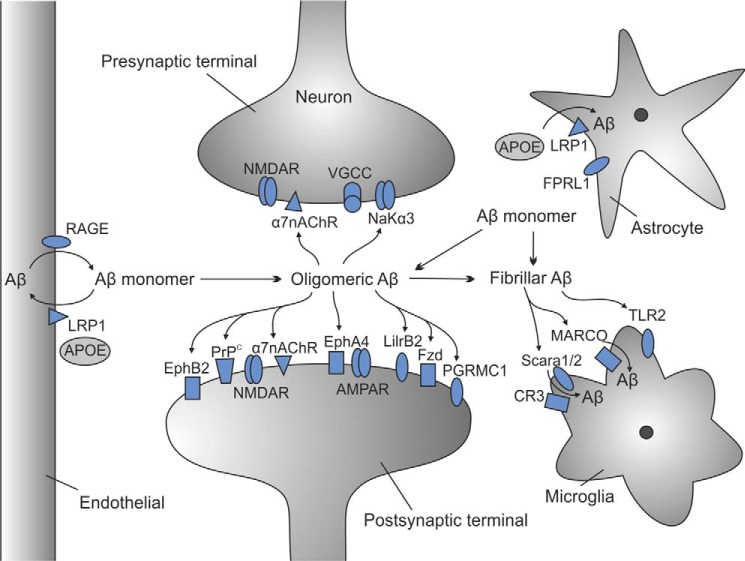
**Aβ receptors and their cellular locations.** Aβ monomers aggregate into oligomers and fibrils in the brain, interacting with a variety of receptors on the presynaptic and postsynaptic membranes of neurons, on endothelial cells, and on astrocytes and microglia. The endothelial receptors RAGE and LRP1 are involved in Aβ monomer clearance through the blood-brain barrier. LRP1 also mediates monomer efflux into astrocytes. The microglial receptors Scara1/2 and MARCO are linked to Aβ clearance by interaction with fibrillar Aβ. Oligomeric Aβ is widely viewed as the pathogenic species, triggering synaptic impairment and cell death following interaction with a range of postsynaptic neuronal receptors, including EphB2, PrP^C^, and α7nAChR, which are linked to NMDAR dysfunction. AβO also bind to EphA4, LilrB2, Frizzled (Fzd), and PGRMC1 receptors, triggering synaptic impairment. In addition, AβO bind to the presynaptic receptors α7nAChR and NaKα3, which are linked to altering presynaptic calcium levels. See text and Table 1 for details.

Alzheimer disease (AD)^2^ is characterized pathologically by the deposition in the brain of the 40–42-amino acid amyloid-β (Aβ) peptide in extracellular plaques and of the microtubule-binding protein tau in intracellular neurofibrillary tangles. The amyloid cascade hypothesis, formulated over 20 years ago, posits that Aβ, derived from the proteolytic processing of the amyloid precursor protein, is the causative agent in AD pathology and that neurofibrillary tangles, cell loss, vascular damage, and dementia follow (1). A recent and critical interpretation of the existing data concluded that aggregated Aβ acts primarily as a trigger of other downstream processes, particularly tau aggregation, which mediate neurodegeneration (2). Understanding the nature of the interaction of Aβ with neurons and other cell types in the brain is key to a complete understanding of the pathogenesis of AD. Furthermore, identifying the proteins involved in the binding of aggregated forms of Aβ and the downstream cytotoxic signaling pathways that are subsequently activated may reveal sites for therapeutic intervention. In this minireview, we provide an overview of the “bad” receptors involved in the binding and cytotoxic action of Aβ, as well as the “good” receptors involved in Aβ metabolism and clearance from the brain (Fig. 1, Table 1). More detailed information on the Aβ receptors and carriers, including the type of Aβ they bind, the cell type they are expressed on, binding partners, and downstream targets, is provided in supplemental Table 1.

**TABLE 1:** **Aβ receptors and carriers** The Aβ receptors and soluble carrier proteins are classified into “good” receptors that promote the clearance or degradation of Aβ, thereby lowering the amount available to form AβO, and into “bad” receptors that mediate the neurotoxic actions of AβO. See text and supplemental Table I for more details. AMPAR, α-amino-3-hydroxy-5-methyl-4-isoxazolepropionic acid receptor; VLDLR, very low-density-lipoprotein receptor.

Aβ receptors and carriers	Aβ type/conformation	Other interactors	Reference
**“Good” receptors**			
α7-Nicotinic acetylcholine receptor (α 7nAChR)	Aβ42 monomer/LMW oligomers (4–24 kDa)		79
Apolipoprotein E (apoE)	Aβ40/42 monomer	LRP1, LDLR	31
Clusterin (ApoJ)	Aβ40 monomer	LRP2	80
Complement receptor type 3 (CR3 or Mac1)	Aβ40/42 fibrillar	SR-A	81
Formyl peptide receptor (FPR1)/formyl-peptide receptor-like 1 (FPRL1)	Aβ42		81
Heparan sulfate proteoglycan (HSPG)	Aβ40/42 monomer	LRP1	82
Low-density lipoprotein receptor (LDLR)	Aβ40/42 monomer	apoE	83
Low-density lipoprotein receptor-related protein 1 (LRP1)	Aβ40/42 monomer	PICALM, apoE	25–28
Macrophage receptor with collagenous structure (MARCO)	Aβ42 monomer	FPRL1	33
Phosphatidylinositol-binding clathrin assembly (PICALM) protein	Aβ40/42 monomer	Clathrin/LRP1	28
Prion protein (PrP^C^)	Aβ40 monomer	LRP1	29
Scavenger receptors (SCARA1/2)	Aβ42 fibrillar		84

**“Bad” receptors**			
α7nAChR	Aβ42 oligomers (4–56 kDa)		85
AMPA receptor	Aβ42 ADDLs (8–40 kDa) A11-negative		86
Amylin 3 receptor (AMY3)	Aβ42 ADDLs (4–96 kDa)		87
apoE	Aβ40/42 oligomers	VLDLR/LRP1	31, 88
β2 adrenergic receptor (β2AR)	Aβ42 dimer	GluR1 (AMPAR)	89
Clusterin (ApoJ)	Aβ42 oligomer (8–200 kDa)		90
Ephrin A4 (EphA4)	Aβ42 oligomers (4–100 kDa)		91
Ephrin B2 (EphB2)	Aβ42 ADDLs (LMW)	NMDAR, PSD95	55, 58
Fcγ receptor IIb (FcγRllb)	Aβ42 ADDLs (LMW)		92
Frizzled (Fzd)	Aβ40/42 ADDLs (12–96 kDa)		93
Insulin receptor	Aβ42 ADDLs (50–100 kDa)		94
Leukocyte immunoglobulin-like receptor B2 (LilrB2)/PirB	Aβ42 ADDLs (50–150 kDa)		95
Na^+^/K^+^-ATPase neuron-specific α3 subunit (NaKα3)	ASPD (128 kDa spheres)		38
Neuroligin-1	Aβ42 A11-positive	PSD95	96
NMDA receptor	Aβ42 ADDLs (12–96 kDa)	PSD95	10, 55, 57
p75 neurotrophin receptor (p75NTR)	Aβ42 ADDLs (LMW)	DR6	97
P/Q-type calcium channels	Aβ42 globulomers		98
PrP^C^	Aβ42 ADDLs (70–250 kDa) OC-positive	mGluR5, LRP1	20, 21, 43, 48
Receptor for advanced glycation end products (RAGE)	Aβ40/42 monomer		99
SCARB2/ CD36	Fibrillar Aβ	TLR-4, TLR-6	100
Sigma-2/PGRMC1	Aβ42 oligomers (50–75 kDa)		74, 77
Toll-like receptor 2 (TLR2)	Aβ42 fibrillar		81

## The Ligand: Multiple Forms of Aβ

Any discussion of receptors has to take into account the properties of the ligand(s) that binds to that receptor. In the case of the ligand Aβ, this is complicated by the fact that it exists in multiple forms from monomers, through dimers, trimers, and oligomers to protofibrils and fibrils that range in size from 4 kDa to assemblies of >100 kDa, and vary in morphology and conformation (3). Aβ oligomers (AβO) appear to be the most neurotoxic species, triggering various processes that underlie AD, including synaptic dysfunction, impairment of long-term potentiation (LTP), Ca^2+^ dysregulation, mitochondrial dysfunction, endoplasmic reticulum stress, and the activation of pro-apoptotic pathways leading to cell death (4, 5). Various oligomeric forms of Aβ have been isolated from human AD brain and from the brains of AD model mice, as well as from cell culture medium, in addition to being produced from preparations of recombinant or synthetic Aβ peptides (6–10). Almost certainly, preparations of AβO, whether isolated from natural sources or produced *in vitro*, are composed of a number of oligomeric species with diverse biophysical and biological properties existing in dynamic equilibrium (6, 11). This dynamic equilibrium complicates studies when attempting to isolate a particular population of oligomers, *e.g.* by size exclusion chromatography, as the resultant “purified” oligomer preparation will remodel to other species as the preparation resets its equilibrium. Therefore, it is not surprising that there is controversy over which is the toxic form of AβO, and indeed whether there is a single toxic entity (6).

The conformation of AβO aggregates has emerged as a useful classification method that is more biologically relevant than size, given that the structural motifs present on the surface of a protein will determine its binding partners and biological activities. Various conformation-specific antibodies that react with AβO have been produced and characterized (reviewed in Ref. 6). Two of the more widely used conformation-specific antibodies are the A11 and OC antibodies (12, 13), which recognize mutually exclusive structural epitopes on a range of amyloid-forming proteins, not just Aβ, independent of primary amino acid sequence. A11 antibodies recognize out-of-register anti-parallel β sheet structures, whereas OC antibodies detect in-register parallel β sheets (14–16).

A recent study (16) classified brain-derived AβO into two types based in part on their reactivity to these conformation-specific antibodies. Type 1 AβO were A11-immunoreactive (also referred to as Aβ*56) and had no temporal, spatial, or structural relationship to amyloid fibrils, whereas type 2 AβO recognized by OC antibodies were related to amyloid fibrils temporally, spatially, and structurally and represented the majority of oligomers generated *in vivo*. The authors concluded that although most of the soluble Aβ in brains with dense core plaques (*e.g.* AD brains) are type 2 AβO, the bulk of these oligomers are rendered functionally innocuous by their effective containment within plaques. In contrast, they suggested that type 1 AβO may be more directly pathogenic in certain brain regions as they are more finely dispersed than the type 2 AβO (16). Further work is required to reconcile these conclusions with the observations that OC reactivity, not A11 reactivity, correlated with the onset and severity of AD in human brain studies (17, 18) and that only OC-positive oligomers correlated with cognitive decline and promoted tau aggregation and phosphorylation in a different transgenic AD mouse model (18).

Another recent study (19) utilized several oligomer-directed quantitative assays, including a high specificity binding assay based on the affinity of certain AβO for the cellular form of the prion protein (PrP^C^) (PrP-ELISA or PLISA) (20), to assay AβO across brain tissue from multiple AD mouse models and human brain samples. The PrP^C^-interacting AβO represented a distinct population of high molecular weight Aβ assemblies that were as accurate as any other predictor of memory impairment in the AD mouse models and human AD patients. Oligomers interacting with PrP^C^ were preferentially recognized by the OC antibody rather than the A11 antibody (21, 22) and thus would appear to correspond to the type 2 AβO (16). Critically, the fraction of PrP^C^-interacting AβO varied greatly between transgenic AD mouse models and likely determines the extent to which PrP^C^-dependent molecular mechanisms contribute to the progression of AD (19). That different transgenic AD mouse models may produce predominantly one (or a few) of the many potential types of AβO present in the human AD brain clearly complicates interpretation of data from the animal models. More work is required to clarify these discrepancies in AβO type and function both between animal models and between the animal models and the human situation. However, the characterization of different AβO species based on antibody or other conformational recognition (*e.g.* PrP^C^ interaction) is a useful criterion with which to help decipher the contribution of particular oligomeric species to AD pathogenesis. *In vivo* it is highly likely that more than one oligomeric species contributes to toxicity, and thus understanding the temporal and spatial distribution of all AβO types in the brain during the initiation and development of AD, as well as knowing their receptors and mechanisms of toxicity, is essential to progress the field. Although AβO have been proposed to cause neurotoxicity through a variety of mechanisms, including direct interaction with lipids resulting in damage to the membrane through, for example, pore formation, or through intracellular accumulation leading to cytotoxicity (23, 24), here we focus on the binding of Aβ to cell surface receptors.

## The “Good” Aβ Receptors

Proteins that bind Aβ (whether monomeric, oligomeric, or fibrillar forms) and reduce the amount available to aggregate into toxic oligomers can in many ways be considered “good” receptors. Such receptors may internalize Aβ into neurons or other cells (*e.g.* microglia) and target it for lysosomal degradation or remove it from the brain by transcytosis across the blood-brain barrier (BBB) (Fig. 1). One such receptor is the low-density lipoprotein receptor-related protein 1 (LRP1), which binds multiple ligands including monomeric Aβ and is abundantly expressed in various brain cell types. LRP1 has been implicated in mediating Aβ transcytosis across the BBB (25), as well as in the uptake and local clearance of Aβ in vascular smooth muscle cells and neurons (26, 27). Recently, the AD genetic risk factor *PICALM*, which encodes the phosphatidylinositol-binding clathrin assembly (PICALM) protein involved in the endocytosis of various cell surface receptors, was reported to influence Aβ clearance across the BBB through regulating the function of LRP1 in brain endothelial cells (28). PrP^C^ has also been linked to Aβ transport across the BBB (29). PrP^C^ on endothelial cells bound monomeric Aβ40, and genetic knock-out or the addition of a competing PrP^C^ antibody blocked the transcytosis of Aβ40 in a process that also required LRP1 (29). The low-density lipoprotein receptor (LDLR) is also implicated in neuronal and astrocytic Aβ uptake and BBB transcytosis of Aβ (30). Although not cell surface receptors, the carriers apolipoprotein (apo) E and clusterin (apoJ) bind soluble Aβ and facilitate its uptake through receptors such as LRP1 or LRP2 and LDLR, thereby reducing the amount of Aβ available to aggregate (31). Microglial cells surrounding Aβ plaques express the scavenger receptors SCARA1 and SCARA2, which have a high affinity for soluble and fibrillar Aβ and mediate phagocytosis and clearance of Aβ from the brain (32). The macrophage receptor with collagenous structure (MARCO) binds Aβ and activates the ERK1/2 signaling pathway, leading to reduced inflammation (33). Collectively, these and other receptors and carriers (Table 1) work together, alongside other mechanisms for degrading or inactivating Aβ in the extracellular environment, such as the Aβ-degrading enzymes neprilysin and insulin-degrading enzyme (34), to maintain Aβ at low, manageable, non-toxic levels in the brain.

## The “Bad” Aβ Receptors

In contrast to the “good” receptors described above that promote the transcytosis of Aβ out of the brain, one mechanism of action of the “bad” receptors is to mediate the uptake of Aβ into the brain across the BBB. The receptor for advanced glycation end products (RAGE), present on endothelial cells, mediates the influx of circulating Aβ (35). RAGE also internalizes Aβ into neurons, promoting its intracellular aggregation and accumulation, leading to rapid activation of p38 MAPK and mitochondrial dysfunction (36). Contributing to the accumulation of Aβ in the brain is apoE4, a well established genetic risk factor for the development of late-onset AD. As well as being involved in modulating the clearance and degradation of Aβ in the brain, apoE also slows the transport of Aβ across the BBB in an isoform-dependent manner, with apoE4 having the greatest effect (37). The detrimental effects of apoE4 are further exacerbated by its ability to bind to and stabilize AβO, slowing down their transition to fibrils (37).

When the first AβO were prepared from synthetic Aβ42 peptide, the now widely used Aβ-derived diffusible ligands (ADDLs), it was observed that their binding to hippocampal neurons was abolished by treating the cells with trypsin (7). This observation, coupled with the low oligomer concentration (5 nm) required for neurotoxicity, indicated that one or more high-affinity protein receptors are responsible for AβO binding and subsequent neurotoxicity. To date, several candidate “bad” Aβ receptors that bind AβO at the cell surface and then trigger a variety of downstream signaling pathways that negatively impact on neuronal function and survival have been described (Table 1; supplemental Table 1) (38–41). The role of several of these receptors in mediating AβO neurotoxicity is controversial or yet to be reproduced. The heterogeneity and dynamic nature of AβO preparations as discussed above undoubtedly have led to difficulties in first identifying a particular receptor and then in corroborating its involvement in different model systems and between different laboratories. The use of different and often poorly characterized preparations of AβO, different toxicity measurements on divergent target cell populations under different conditions, and the use of different transgenic AD mouse models at different stages of disease all confuse the picture. The recent report that the proportion of PrP^C^-interacting AβO varies between different mouse models of AD (19) may go some way to explain these discordant observations. Indeed this highlights a fundamental issue in the field; it is very unlikely that all receptors bind to the same oligomeric species of Aβ, and binding of different AβO to an individual receptor may be differentially influenced by other receptors or co-receptors in their vicinity (see below). Many of the signaling pathways initiated by these receptors converge into common downstream targets that are ultimately responsible for neurotoxicity and cell death.

## Dynamic Signaling Platforms Mediate AβO Binding and Action

Various lines of evidence suggest that AβO binding to neurons may involve multi-protein cell surface receptor complex(es) whose assembly is initiated upon binding of oligomers to one or more of the receptor proteins listed in Table 1. These signaling platforms or signalosomes (5, 39, 42) will be formed from complexes of proteins and lipids in the plane of the plasma membrane, and will be transient in nature and likely involved in both physiological and pathological responses, contributing to both neuroprotection and neurotoxicity. The relative contribution to these two endpoints may depend on multiple factors, including the type and concentration of oligomer species, the compartmentalization of particular receptors and signaling effectors into different signaling platforms, the relative local interaction and concentration of particular receptors, co-receptors, and lipids, the interplay between the various downstream signaling pathways, and the rate of receptor down-regulation/internalization.

**FIGURE 2.**
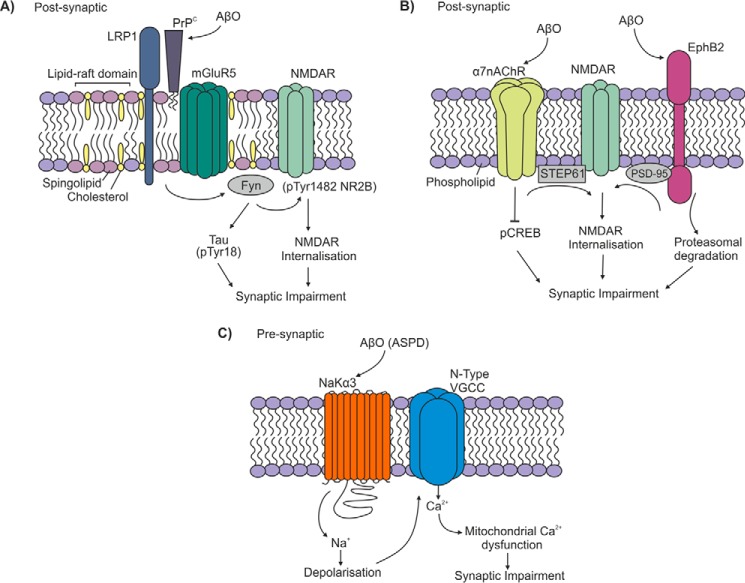
**Aβ oligomer receptor signaling platforms.** AβO induce synaptic impairment and neuronal cell death by interacting with multiple receptor signaling platforms. *A*, PrP^C^-based, cholesterol- and sphingolipid-rich lipid raft signaling platform. The co-receptors LRP1 and mGluR5 cluster with PrP^C^ upon AβO binding and lead to activation of Fyn kinase, which phosphorylates NMDAR and tau. *pTyr18*, phospho-Tyr-18; *pTyr1482*, phospho-Tyr-1482. *B*, both α7nAChR and EphB2 bind AβO and induce NMDAR-mediated dysfunction and synaptic impairment. *pCREB*, phospho-cAMP-response element-binding protein. *C*, the presynaptic NaKα3 binds ASPD oligomers, inducing Ca^2+^ influx via N-type VGCCs, resulting in mitochondrial dysfunction, tau phosphorylation, and synaptic impairment.

One such signaling platform is based on PrP^C^ (Fig. 2*A*). PrP^C^ was identified to bind AβO, but not monomers or fibrils, with high affinity (*K_d_* ∼0.4 nm) (43, 44) and to selectively interact with high molecular mass assemblies of AβO in AD but not control brains (45). PrP^C^ was responsible for the AβO-mediated inhibition of LTP in hippocampal slices (43) and was also required for the manifestation of memory impairments in an AD mouse model (46). AβO binding to PrP^C^ leads to activation of Fyn kinase, which in turn phosphorylates the GluN2B subunit of *N*-methyl-d-aspartate receptors (NMDARs), which was coupled to an initial increase and then a loss of surface NMDARs (20). In addition, the AβO activation of Fyn leads to tau phosphorylation (47). Both mGluR5 (48) and LRP1 (21) have been identified as co-receptors required for the PrP^C^-bound AβO to activate Fyn (Table 1).

PrP^C^ localizes to cholesterol- and sphingolipid-rich, detergent-resistant lipid rafts due to the saturated acyl chains in its glycosylphosphatidylinositol anchor and to an N-terminal targeting signal interacting with the heparan sulfate proteoglycan, glypican-1 (49, 50). PrP^C^ has been proposed as a key scaffolding protein for the dynamic assembly of cell surface signaling modules (51), and PrP^C^, along with the microdomain-forming flotillin or caveolin proteins, may lead to the local assembly of membrane protein complexes at sites involved in cellular communication, such as cell-cell contacts, focal adhesions, the T-cell cap, and synapses (52). The integrity of lipid rafts is critical for the cell surface binding of AβO and the subsequent activation of Fyn. Treatment of cells with methyl-β-cyclodextrin, which depletes cellular cholesterol and thus disrupts the cholesterol-rich lipid rafts, caused the re-localization of PrP^C^ and Fyn from detergent-resistant rafts to detergent-soluble, non-raft regions of the membrane (21). Surprisingly, disruption of the rafts with methyl-β-cyclodextrin significantly reduced (by >80%) the cell surface binding of the AβO, although the cell surface expression of PrP^C^ was unaffected, and prevented the AβO from activating Fyn (21). The addition of AβO to neurons caused a large increase of mGluR5 in the detergent-resistant fraction (53), and on binding oligomers, the co-localization of LRP1 and PrP^C^ increased (21), suggesting that binding of AβO to PrP^C^ causes these co-receptors to cluster together in rafts and activate the signaling complex. This cell-surface, raft-based signaling complex based on PrP^C^ may be key in mediating the neurotoxic actions of type 2 AβO (Fig. 2*A*). Another cholesterol-rich, raft-based platform may involve the presynaptic α7-nicotinic acetylcholine receptor (α7-nAChR) as its AβO-mediated activation was attenuated on disruption of the rafts by cholesterol depletion (54).

Another signaling platform(s) is likely based on NMDARs (Fig. 2*B*), which are necessary but not sufficient for AβO binding (reviewed in Ref. 5). NMDARs are anchored by PSD95, which acts as a scaffold to organize multiple membrane-associated proteins at synapses and which interacts with other AβO receptors including EphB2 (55). Binding of AβO to postsynaptic density complexes containing NMDARs promoted dendritic spine loss in an NMDAR-dependent manner and abolished NMDAR-dependent LTP (55, 56). Antibodies against the subunits of NMDAR blocked the binding of AβO to neurons, and the NMDAR antagonist Memantine completely protected against AβO-induced reactive oxygen species formation (57), indicating that the receptors are required for binding and downstream action of AβO. However, no direct binding of AβO to NMDAR subunits has been reported. The EphB2 receptor modulates NMDAR by tyrosine phosphorylation and recruits active NMDAR to excitatory synapses. AβO interacted directly with the extracellular fibronectin repeats of EphB2, which led to depletion of surface EphB2 by enhancing its proteasomal degradation and to the internalization of GluN1 subunit-containing NMDARs (58). The α7-nAChR also induces AβO-mediated NMDAR dysfunction and synaptic impairment. α7-nAChR binds AβO with high affinity, and binding leads to activation of the channel, increased cytosolic Ca^2+^, and subsequent activation of protein phosphatase 2B (PP2B). De-phosphorylation of the tyrosine phosphatase striatal-enriched protein tyrosine phosphatase (STEP) via PP2B promotes STEP to dephosphorylate Tyr-1472 on the NMDAR subunit GluN2B, thereby disrupting its binding to PSD95, ultimately leading to the internalization of the receptor (59). Thus, binding of AβO (possibly distinct species) to multiple receptors promotes neurotoxicity via NMDAR. Although both EphB2 and α7-nAChR mediate AβO action via NMDARs, whether they are located in the same signaling platform awaits to be determined. Although binding of AβO to PrP^C^ also results in altered NMDAR function, EphB2 does not link AβO-PrP^C^ complexes to Fyn activation (48), providing clear evidence for the existence of distinct AβO-binding signaling platforms.

A further signaling platform is based on the Na^+^/K^+^-ATPase (Fig. 2*C*), whose neuron-specific α3 subunit (NaKα3) was recently identified to bind amylospheroids (ASPD) (38). ASPD are 15-nm spherical AβO that are distinct from ADDLs, that are not recognized by the A11 conformation-dependent antibody, and that caused selective degeneration of mature human neurons (60). The direct binding of ASPD to NaKα3 impaired its activity, resulting in an increase in cytoplasmic Na^+^ and depolarization of the neuron. This in turn activated N-type voltage-gated Ca^2+^ channels (N-type VGCC), leading to Ca^2+^ overload in the cytoplasm and mitochondria, ultimately leading to tau phosphorylation and degeneration of neurons. This signaling platform is localized on the presynaptic membrane (Fig. 1).

Further signaling platforms, based on other groupings of the receptors in Table 1, possibly in association with distinct combinations of membrane lipids, may be involved in binding the same and/or other oligomeric forms of Aβ and transducing neurotoxic signals. It should also be noted that although we have described these different signaling platforms as distinct entities (Fig. 2), it is possible that they are not structurally or functionally isolated and that “super” platforms exist which contain multiple receptors interacting with multiple oligomeric forms of Aβ. Furthermore, the protein and lipid composition of these dynamic signaling platforms may alter as a result of electrophysiological activity, oxidative damage, changes in lipids such as reduced cholesterol, hypoxia, and other cellular activities, and insults that are known to influence the initiation and/or progression of AD, thus influencing AβO binding and the downstream signaling pathways that are activated.

Does the AβO-promoted clustering of receptors lead to the induction of aberrant neurotoxic signaling (53) or over-stimulation of a physiological pathway (for example, due to prolonged stabilization of an otherwise transient complex involved in normal signaling processes), *i.e.* gain of toxic function? Or is it the hijacking of the signaling platform by AβO that disrupts normal physiological signaling, *i.e.* loss of function? Or a combination of these that leads to the neurotoxicity apparent in AD? The amount and/or activity of, and interactions between, individual signaling platform components are likely finely balanced. Either an increase or a decrease in a particular component or an alteration in the interaction between components may be sufficient for AβO to trigger neurotoxicity. It is possible that it is the binding of different AβO to multiple signaling platforms that initiates the complex series of events underlying AD. Following on from this, in the transgenic mouse models that predominantly produce only one type of AβO (19), not all of these signaling platforms will be engaged, resulting in activation of only some of the downstream signaling pathways and thus not recapitulating the complete array of molecular, cellular, and pathological responses seen in the human disease.

## Therapeutic Approaches to Blocking AβO Action

**FIGURE 3.**
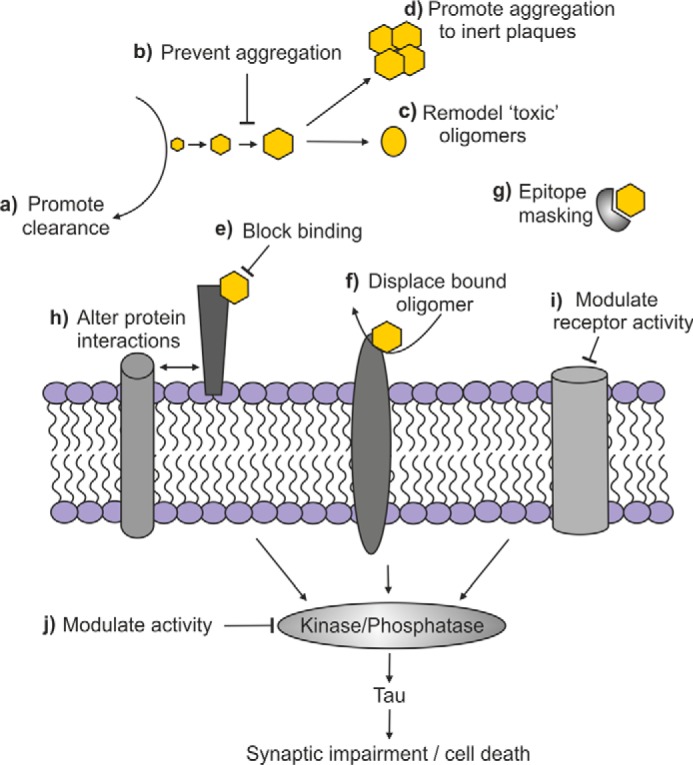
**Potential targets for therapeutic intervention in Aβ oligomer receptor signaling platforms.** The toxic actions of AβO can be prevented by multiple approaches. Their accumulation can be prevented by promoting the clearance/degradation of Aβ monomers (*a*), preventing aggregation (*b*), remodeling “toxic” conformations (*c*), or promoting aggregation to inert fibrils or plaques (*d*). AβO action at the cell surface can be targeted by blocking their binding to receptors (*e*), displacing bound AβO (*f*), or masking the epitope on the oligomers to prevent binding to their receptor (*g*). The receptors themselves can be targeted either by preventing aberrant clustering of receptors mediated by AβO (*h*) or by allosterically modulating receptor activity (*i*). Downstream signaling of AβO can be targeted by modulating kinase/phosphatase activity (*j*) in the downstream signal transduction pathways. See text for specific examples of each.

AβO and their cell surface receptors provide a multitude of potential therapeutic targets (Fig. 3). For example, the accumulation of the “toxic” AβO could be prevented by blocking their formation, promoting their aggregation into larger order “inert” fibrils or plaques, altering their conformation, or inducing their clearance or degradation (Fig. 3, *a–d*). Immunotherapy is being actively explored as a potential means to reduce Aβ levels in the brain, although results from several clinical trials have been disappointing (61). Whether natural antibodies or other antibody preparations that bind to conformational epitopes on AβO and are therefore selective for AβO over other forms of Aβ will be more effective than antibodies that recognize peptide epitopes and thus bind both monomeric and oligomeric forms of Aβ awaits to be seen (62–64). The polyphenols, resveratrol and (−)epigallocatechin-gallate (EGCG) convert soluble AβO into non-toxic aggregates (65, 66) whose binding to PrP^C^ is severely impaired and which no longer activate Fyn (21). Another natural compound, brazilin, has recently been identified to potently remodel mature fibrils, preventing the formation of toxic oligomers by secondary nucleation (67). As clearance of Aβ across the BBB may be impaired in AD (68), approaches to increase transcytosis of Aβ out of the brain may hold potential. In this respect, a soluble form of LRP1 promoted Aβ clearance in a transgenic AD mouse model (69).

Another approach is to prevent the initial interaction of AβO with its receptor or to displace AβO that are already bound (Fig. 3, *e* and *f*). Following identification of PrP^C^ as a high affinity receptor for AβO (43), immuno-targeting of PrP^C^ was shown to block completely the LTP impairments caused by AβO derived from human AD brain extracts (70, 71), and intra-cerebral infusion of an anti-PrP^C^ monoclonal antibody reversed the memory impairments in a transgenic AD mouse model (72). Recently, the small molecule Chicago Sky Blue 6B was identified in a high-throughput screen to bind to PrP^C^ and inhibit AβO binding (73). Sigma-2/PGRMC1 was identified as a receptor mediating the binding and toxicity of both brain-derived and synthetically prepared AβO following the screening of a library of CNS drug-like small molecules that blocked AβO-induced deficits (74). The compounds identified were ligands for Sigma-2/PGRMC1 and prevented AβO from binding to primary hippocampal neurons and also displaced bound oligomeric species. The small molecule rhynchophylline was identified as a novel inhibitor of EphA4, which blocked the ligand-binding domain of EphA4 and rescued AβO-induced deficits (75). Surface epitope masking peptides have recently been shown to prevent ASPD interacting with NaKα3 (38) (Fig. 3*g*). Tetrapeptides mimicking the binding region of this receptor bound to the surface of ASPD, subsequently blocking their interaction with the receptor and preventing ASPD-induced impairments but without affecting the normal function of the Na^+^/K^+^ATPase (38).

Complete blocking of receptors may have deleterious effects on neuronal function; however, modulating receptor activity is another potential approach to abrogate AβO action (76) (Fig. 3*h*). Antagonism of the mGluR5 receptor using negative allosteric modulators prevented AβO-induced spine loss and cognitive deficits in transgenic mice (48). The Sigma-2/PGRMC1 ligands also acted as allosteric antagonists for the receptor, preventing aberrant signaling, as well as the subsequent spine loss and cognitive impairments in AD transgenic mice (77). Another approach is to target the downstream signal transduction pathways activated upon AβO binding to its receptors (Fig. 3*i*). For example, given that AβO binding to PrP^C^ activates Fyn, a Phase 1b trial of a potent small molecule inhibitor of Src and Fyn for the treatment of AD is underway (78). Ultimately, a combined therapeutic approach, targeting more than one AβO species, its receptor(s), and/or its downstream signaling pathway, will likely be required to alleviate all the neurotoxic effects of the multiple oligomeric forms of Aβ.

## Concluding Remarks

Although much progress has been made in identifying Aβ receptors, several questions remain unanswered. How many distinct AβO receptors and signaling platforms are there? What is the contribution of each receptor and signaling platform to AβO-mediated toxicity? What are the individual components in each signaling platform, and how do their compositions, as well as the interactions between them, differ between AD and healthy individuals? Are different AβO signaling platforms involved depending on the initial trigger of disease, and what is their spatial and temporal contribution to disease pathogenesis? Given that there are multiple species of AβO, and that some transgenic mouse models appear to have predominantly one type of AβO, each interacting with a distinct set of receptors, what is the most appropriate animal model? How can we target specific signaling platforms for therapeutic intervention in AD without disrupting the normal physiological roles of these signaling complexes? Answers to these questions will come only from further experimental work comparing the binding of defined AβO preparations (characterized on the basis of biophysical and conformational properties) with each of the identified receptors *in situ* on cells and *in vivo* in appropriate animal models. However, the recognition that there are multiple Aβ receptors, binding different forms of Aβ, possibly preferentially in different stages in the development of AD, provides several opportunities for therapeutic intervention as highlighted here. What must also be recognized is that not only are there “bad” Aβ receptors binding oligomeric forms of Aβ and triggering cytotoxicity, but there are also “good” receptors involved in Aβ clearance and metabolism, as well as some like PrP^C^ that may play dual roles.

## Supplementary Material

Supplemental Data

## Footnotes

2 The abbreviations used are:

AD: Alzheimer disease
Aβ: amyloid-β
AβO: amyloid-β oligomer(s)
α7-nAChR: α7-nicotinic acetylcholine receptor
ADDL: Aβ-derived diffusible ligand
apo: apolipoprotein
ASPD: amylospheroid(s)
BBB: blood-brain barrier
Eph: Ephrin
LDLR: low-density lipoprotein receptor
LMW: low molecular weight
LRP1: low-density lipoprotein receptor-related protein 1
LTP: long-term potentiation
NaKα3: Na^+^/K^+^-ATPase neuron-specific α3 subunit
NMDAR: *N*-methyl-d-aspartate receptor
PrP^C^: cellular prion protein
RAGE: receptor for advanced glycation end products
mGluR: metabotropic glutamate receptor.
