# Supplementary material for: Amyloid-β Receptors: The Good, the Bad, and the Prion Protein
Source: J Biol Chem. 2015 Dec 30;291(7):3174–83. doi: 10.1074/jbc.R115.702704 (PMC4751366; doi:10.1074/jbc.R115.702704)
Supplement: Supplemental Data [file supp_291_7_3174__index.html]

Amyloid-β receptors: the good, the bad and the prion protein — Amyloid-β Receptors: The Good, the Bad, and the Prion Protein — MINIREVIEW: Aβ Receptors — Supplemental Data 

# Amyloid-β Receptors: The Good, the Bad, and the Prion Protein

## Supplemental Data

- Supplemental Table 1 (.pdf, 222 KB) - Supplemental Table 1
