# Supplementary material for: Amyloid-β Receptors: The Good, the Bad, and the Prion Protein
Source: J Biol Chem. 2015 Dec 30;291(7):3174–83. doi: 10.1074/jbc.R115.702704 (PMC4751366; doi:10.1074/jbc.R115.702704)
Supplement: Supplemental Data [file 10.1074_R115.702704_jbc.R115.702704-1.pdf]

Amyloid-β receptors: the good, the bad and the prion protein

Heledd H. Jarosz-Griffiths, Elizabeth Noble, Jo V. Rushworth and Nigel M. Hooper

Supplementary Table 1. Aβ receptors and carriers

Receptor	Aβ Type/ conformation	Cell type	Binding	Downstream target following Aβ binding	Other interactors
**"Good" receptors**					
α7-nicotinic acetylcholine receptor (α7nAChR)	Aβ40/42 monomer/ LMW oligomers (4-24kDa)	Neurons, astrocytes, microglia	Aβ42 (200 pM) activate channel (1). Aβ42 12-28 binds to agonist binding site of α7nAChR (2).	Modulates hippocampal synaptic plasticity by activating pre-synaptic α7nAChR and enhancing cognitive function in mice (1).	
Apolipoprotein E (apoE)	Aβ40/42 monomer	Astrocytes, microglia	Kd = 48.1nM E2; Kd = 63.7nM E3; Kd = 75.9nM E4 (3).	apoE stabilizes Aβ monomers, facilitates uptake via co-receptors and enhances degradation of Aβ (4) (reviewed in (5)).	LRP1 and LDLR (reviewed in (5)).
Clusterin (apoJ)	Aβ40 monomer	Synthesised by neurons, astrocytes,	Aβ40 Kd = 4.8nM Binds (8kDa - 200kDa) and forms stable complexes (6).	Binds soluble Aβ in CSF/plasma. LRP2 mediates the internalisation and degradation of the clusterin/Aβ complex (6,7).	LRP2(6)
Complement receptor type 3 (CR3 or Mac1)	Aβ40/42 fibrillar	Microglia	Co-localisation of CR3 with fibrillar Aβ40 (8).	Binds and internalises synthetic fibrillar Aβ and human AD amyloid cores (9). Scavenger receptor A (SR-A) may act in parallel to induce Aβ phagocytosis (9).	SR-A (8)
Formyl peptide receptor (FPR1) and Formyl-peptide receptor-like 1 (FPRL1)	Aβ42	Microglia, astrocytes	Direct binding of Aβ42 monomer to N-terminus, 4th transmembrane domain and 3rd extracellular loop (10).	Aβ42-FPRL1 complex is internalised into HEK293 cells expressing FPRL1 and macrophages (11).	
Heparan sulphate proteoglycan (HSPG)	Aβ40/42 monomer	Neurons	Binds Aβ at residues 13-16 (12).	HSPG binds Aβ, and mediates endocytosis via LRP1 (12).	LRP1(12)
Low-density lipoprotein receptor (LDLR)	Aβ40/42 monomer	Astrocyte, endothelial, neurons	Aβ40/42 Kd = 47.4/ 37.4nM; Binding disrupted with PCSK9 and RAP (13,14).	LDLR regulates the cellular uptake and metabolism of Aβ as well as BBB transcytosis (13,14).	apoE(13)

Low-density lipoprotein receptor-related protein 1 (LRP1)	Aβ40/42 monomer	Endothelial, vascular smooth muscle cells, neurons	Co-localisation of LRP1 with Aβ40/42. LRP1 binds to Aβ40 > Aβ42 (15).	LRP1 regulates Aβ clearance across BBB (16), and local clearance into different cell types (17,18).	PICALM, apoE (16)
Macrophage receptor with collagenous structure (MARCO)	Aβ42 monomer	Microglia, astrocytes	Synthetic Aβ42, MARCO agonist fucodian inhibits Aβ signal transduction (19).	Forms a complex with Aβ/FPRL1. FRLP1 signalling in microglia via ERK 1/2, and inhibition of cAMP leads to decreased inflammatory response (19).	FPRL1 (19)
Phosphatidylinositol-binding clathrin assembly protein (PICALM)	Aβ40/42 monomer	Endothelial	Co-localisation of LRP1: PICALM complex with Aβ40/42 (20)	Regulates PICALM/clathrin-dependent internalization of Aβ bound to the LRP1 leading to Aβ transcytosis and clearance (20).	Clathrin, LRP1(20)
Prion protein (PrP^C)	Aβ40 monomer	Endothelial	[125I]-Aβ40 binding to recombinant PrP (pM range) (21).	Required for Aβ40 transcytosis across the BBB (21).	LRP1(21)
Scavenger receptor A (SCARA1/2 or SR-A)	Aβ42 fibrillar	Microglia, astrocytes	Synthetic Aβ42 (6-9nm fibrils) (22).	Microglia bind and phagocytose Aβ via SCARA1 (22).	
**“Bad” receptors**					
α7nAChR	Aβ40/42 oligomers (8-56kDa)	Neurons, astrocytes, microglia	Direct binding of Aβ40/42 to agonist binding site of α7nAChR	ADDLs bind and activate α7nAChR, increasing Ca^{2+} and leading to NMDAR endocytosis via PP2B (23). ADDL binding can also lead to ERK/MAPK pathway activation (reviewed in (24)).	
α-amino-3-hydroxy-5-methyl-4-isoxazolepropionic acid receptor (AMPAR)	Aβ42 ADDLs (8-40kDa) A11 negative	Neurons	ADDLs bind to GluR2 but not GluR1. ACU954 antibody blocked ADDL binding to AMPAR (25).	ADDLs bind GluR2-containing synapses, where they cause the removal of surface AMPARs via a PP2B/calcineurin dependent endocytosis mechanism leading to synaptic loss (25).	
Amylin 3 receptor (AMY3)	Aβ42 ADDLs (4-96kDa)	Human embryonic kidney 293 (HEK293) cells	Aβ42 activate AMY3 expressing HEK293 cells.	Aβ42 increases cytosolic cAMP and Ca^{2+} trigger the signal transduction mediators protein kinase A, MAPK, Akt, and cFos.	(26)

apoE	Aβ40/42 oligomers	Astrocytes, microglia	Affinity for Aβ oligomers E4 > E3 > E2. apoE stabilises Aβ oligomers that are in a different orientation (27).	Slows the transport of Aβ across the BBB in an isoform-dependent manner (E4 > E3 > E2) (5). apoE4-AβO complex is redirected to VLDLR and is cleared more slowly (28).	VLDLR/ LRP1 (28)
β2 adrenergic receptor (β2AR)	Aβ42 dimer	Neurons	Direct binding Aβ42 dimer to N-terminal β2AR.	Initiates the β2AR cAMP/ PKA signalling pathway leading to phosphorylation of GluR1 subunit of AMPAR, enhancing receptor activation.	GluR1 (AMPAR) (29)
Clusterin (ApoJ)	Aβ42 oligomer (8-200kDa)	Neurons	Based on Aβ40 Kd = 4.8nM Binds and forms stable complexes (6).	Aβ oligomer increased intracellular clusterin resulting in the p53-dependent induction of DKK1. DKK1 interacts with LRP5/6 and prevents interaction with frizzled leading to activation of wnt-PCP-JNK pathway (30).	
EphA4	Aβ42 oligomers (4-80kDa) AFM: 20nm	Neurons	Aβ42 oligomers bind to cells overexpressing EphA4. Inhibited by EphA4 antagonist (KYL)(31).	Aβ42 oligomers cause re-localisation of EphA4 from dendritic spines to shaft, leading to cAbl-mediated LTP blockage and reduction in dendritic spine number (31).	
EphB2	Aβ42 LMW ADDLs	Neurons	Aβ42 oligomers bind to fibronectin-type III repeat domain of EphB2(32).	ADDL binding promotes internalisation and proteasomal degradation of EphB2 in neuronal cultures. Loss of EphB2 leads to a decrease in surface GluN1 of NMDAR (32,33).	NMDAR, PSD95 (32).
Fcγ receptor IIb (FcγRllb)	Aβ42 ADDLs LMW.	Neurons	Aβ42 oligomers Kd = 56.7nM. N-terminus of Aβ42 predicted to bind to the Ig-like domain 2 of FcγRIIb(34).	Aβ42 oligomers mediate FcγRIIb homo-aggregation and induce JNK-mediated signalling leading to decreased LTP and spine density (34).	

Frizzled	Aβ40/42 (ADDLs 12kDa-96kDa)	Neurons	Aβ40 binding to Frizzled cysteine-rich domain (Fz5CRD): Kd = ~105nM (35).	Aβ oligomer binding to Fz5CRD inhibits the the canonical Wnt signaling pathway by preventing wnt binding and recruitment of dishevelled (35).	
Insulin receptor	Aβ42 ADDLs (50-100kDa)	Neurons	Probable Aβ42 binding site (residues 16-25) shares common recognition motif with residues 21-30 of the β-chain of insulin (36).	ADDLs downregulate cell surface levels of insulin receptors, mediated by CK2 and CaMKII(37). Insulin competitively blocks ADDL binding (36).	
LilrB2/PirB	Aβ42 ADDLs HMW (50-150kDa)	Neurons	Aβ42 oligomers Kd = 206 nM LilrB2. N-terminal Ig domains (D1D2) of PirB and of LilrB2 are critical for binding (38).	Aβ42 oligomer/PirB complex recruit cofilin signaling modules (PP2A and PP2B/calcineurin) to facilitate actin depolymerization, resulting in synaptic loss (38).	
Na^+/K^+-ATPase neuron-specific α3 subunit (NAKα3)	Amylospheroids (ASPD) (128 kDa) 10-15nM spheres in TEM.	Neurons	ASPD Kd = 28.6 nM, A11 negative (39).	ASPD directly bind to NAKα3, impair its activity leading to Ca^{2+} dyshomeostasis, increased tau phosphorylation and neuronal loss. ASPD binding peptides inhibited the ASPD-deficits (39).	
Neuroligin-1 (NL1)	Aβ42, A11 positive	Neurons	Aβ42 Kd = ~250 nM. Co-localise with NL1 in hippocampal neurons (40).	NL1 stimulates the formation of Aβ oligomers and targeting of Aβ oligomers to postsynaptic regions of excitatory synapses (40).	PSD95
N-methyl-D-aspartate receptor (NMDAR)	Aβ42 ADDLs (12-96kDa)	Neurons	Aβ oligomers bind to synaptosomes (containing PSD95, GluN1 and GluN2A/B) Co-IP with anti-ADDL antibody. Knockdown of GluN1 reduced ADDL binding to neurons (33).	Loss of NMDAR following ADDL binding leads to change in spine morphology and loss, and decreases in LTP (33,41,42)	PSD95 (33)

p75 neurotrophin receptor (p75NTR)	Aβ42 oligomer LMW ADDLs	Neurons	Aβ oligomers interact with the extracellular domain of p75NTR (43,44).	Aβ oligomers binds to p75NTR, promoting an interaction with DR6 and downstream activation of JNK-pathway leading to neuronal death (43,44).	DR6
P/Q-type calcium channels	Aβ42 globulomers	Neurons	Aβ42 globulomers impair P/Q-type calcium currents, reversed with roscovitine agonist.	Aβ42 globulomer strongly impairs presynaptic P/Q-type calcium currents at both glutamatergic and GABAergic synapses	(45)
PrP^C	Aβ42 oligomers (70-250kDa) OC positive	Neurons	Aβ42 oligomers Kd = 0.4nM for neuron cultures. PrP^C binding sites: 23–27, 92–110 (46)	High affinity receptor for Aβ42 oligomers leading to LTP impairment in AD mouse models. Binding of Aβ oligomers activates Fyn kinase, induce phosphorylation of GluN2B leading to transient overexpression of NMDAR on the surface leading to spine loss (47-51).	mGluR5 (49), LRP1 (47)
Receptor for advanced glycation end products (RAGE/ sRAGE)	Aβ40/42 Monomer	Endothelial cells, microglia, neurons	Aβ40 Kd = 55 nM	Mediates Aβ transport across BBB into brain (52). Microglial RAGE signals through p38 MAPK/JNK pathway leading to synaptic dysfunction through JNK-mediated IL-β release (53).	
SCARB2/ CD36	Fibrillar Aβ	Microglia		Aβ binds CD36 and signals via Fyn/ Lyn, activating MAPK pathways leading to ROS production and inflammasome activation(54,55)	TLR-4, TLR-6 (55)
Sigma-2/PGRMC1	Aβ42 oligomers (50-75kDa)	Neurons, Microglia	Aβ42 oligomers Kd = 518 nM for neuronal cultures (56)	Aβ42 oligomers increase Sigma-2 levels and causes deficits in membrane trafficking. CogRx molecules and antibodies specific for the C-terminal region of Sigma-2 prevent and competitively	

				displace oligomer binding (56,57).
Toll-like receptor 2 (TLR2)	Aβ42 fibrillar	Microglia, astrocytes	Co-localisation of Aβ42 with TLR2. Binding mediated via N-terminal ectodomain of TLR2 (58).	Knockdown of TLR2 reduces expression of inflammatory molecules and an increase in Aβ internalisation by phagocytosis (58).

References:

1. Puzzo, D., Privitera, L., Leznik, E., Fa, M., Staniszewski, A., Palmeri, A., and Arancio, O. (2008) Picomolar amyloid-beta positively modulates synaptic plasticity and memory in hippocampus. *J. Neurosci.* **28**, 14537-14545
2. Espinoza-Fonseca, L. M. (2004) Base docking model of the homomeric alpha7 nicotinic receptor-beta-amyloid(1-42) complex. *Biochem. Biophys. Res. Commun.* **320**, 587-591
3. Yamauchi, K., Tozuka, M., Nakabayashi, T., Sugano, M., Hidaka, H., Kondo, Y., and Katsuyama, T. (1999) Higher avidity binding of apolipoprotein (E-AII) complex than of apolipoprotein E monomer to beta-amyloid. *J. Neurosci. Res.* **58**, 301-307
4. Jiang, Q., Lee, C. Y., Mandrekar, S., Wilkinson, B., Cramer, P., Zelcer, N., Mann, K., Lamb, B., Willson, T. M., Collins, J. L., Richardson, J. C., Smith, J. D., Comery, T. A., Riddell, D., Holtzman, D. M., Tontonoz, P., and Landreth, G. E. (2008) ApoE promotes the proteolytic degradation of Abeta. *Neuron* **58**, 681-693
5. Liu, C. C., Kanekiyo, T., Xu, H., and Bu, G. (2013) Apolipoprotein E and Alzheimer disease: risk, mechanisms and therapy. *Nat. Rev. Neurol.* **9**, 106-118
6. Hammad, S. M., Ranganathan, S., Loukinova, E., Twal, W. O., and Argraves, W. S. (1997) Interaction of apolipoprotein J-amyloid beta-peptide complex with low density lipoprotein receptor-related protein-2/megalin. A mechanism to prevent pathological accumulation of amyloid beta-peptide. *J. Biol. Chem.* **272**, 18644-18649
7. Narayan, P., Meehan, S., Carver, J. A., Wilson, M. R., Dobson, C. M., and Klenerman, D. (2012) Amyloid-beta oligomers are sequestered by both intracellular and extracellular chaperones. *Biochemistry* **51**, 9270-9276
8. Strohmeyer, R., Ramirez, M., Cole, G. J., Mueller, K., and Rogers, J. (2002) Association of factor H of the alternative pathway of complement with agrin and complement receptor 3 in the Alzheimer's disease brain. *J. Neuroimmunol.* **131**, 135-146
9. Fu, H., Liu, B., Frost, J. L., Hong, S., Jin, M., Ostaszewski, B., Shankar, G. M., Costantino, I. M., Carroll, M. C., Mayadas, T. N., and Lemere, C. A. (2012) Complement component C3 and complement receptor type 3 contribute to the phagocytosis and clearance of fibrillar Abeta by microglia. *Glia* **60**, 993-1003
10. Le, Y., Ye, R. D., Gong, W., Li, J., Iribarren, P., and Wang, J. M. (2005) Identification of functional domains in the formyl peptide receptor-like 1 for agonist-induced cell chemotaxis. *FEBS J.* **272**, 769-778
11. Yazawa, H., Yu, Z. X., Takeda, Le, Y., Gong, W., Ferrans, V. J., Oppenheim, J. J., Li, C. C., and Wang, J. M. (2001) Beta amyloid peptide (Abeta42) is internalized via the G-protein-coupled receptor FPRL1 and forms fibrillar aggregates in macrophages. *FASEB J.* **15**, 2454-2462
12. Kanekiyo, T., Zhang, J., Liu, Q., Liu, C. C., Zhang, L., and Bu, G. (2011) Heparan sulphate proteoglycan and the low-density lipoprotein receptor-related protein 1 constitute major pathways for neuronal amyloid-beta uptake. *J. Neurosci.* **31**, 1644-1651
13. Basak, J. M., Verghese, P. B., Yoon, H., Kim, J., and Holtzman, D. M. (2012) Low-density lipoprotein receptor represents an apolipoprotein E-independent pathway of Abeta uptake and degradation by astrocytes. *J. Biol. Chem.* **287**, 13959-13971

14. Kim, J., Castellano, J. M., Jiang, H., Basak, J. M., Parsadanian, M., Pham, V., Mason, S. M., Paul, S. M., and Holtzman, D. M. (2009) Overexpression of low-density lipoprotein receptor in the brain markedly inhibits amyloid deposition and increases extracellular A beta clearance. *Neuron* **64**, 632-644
15. Deane, R., Wu, Z., Sagare, A., Davis, J., Du Yan, S., Hamm, K., Xu, F., Parisi, M., LaRue, B., Hu, H. W., Spijkers, P., Guo, H., Song, X., Lenting, P. J., Van Nostrand, W. E., and Zlokovic, B. V. (2004) LRP/amyloid beta-peptide interaction mediates differential brain efflux of Abeta isoforms. *Neuron* **43**, 333-344
16. Shibata, M., Yamada, S., Kumar, S. R., Calero, M., Bading, J., Frangione, B., Holtzman, D. M., Miller, C. A., Strickland, D. K., Ghiso, J., and Zlokovic, B. V. (2000) Clearance of Alzheimer's amyloid-ss(1-40) peptide from brain by LDL receptor-related protein-1 at the blood-brain barrier. *J. Clin. Invest.* **106**, 1489-1499
17. Kanekiyo, T., Cirrito, J. R., Liu, C. C., Shinohara, M., Li, J., Schuler, D. R., Shinohara, M., Holtzman, D. M., and Bu, G. (2013) Neuronal clearance of amyloid-beta by endocytic receptor LRP1. *J. Neurosci.* **33**, 19276-19283
18. Kanekiyo, T., Liu, C. C., Shinohara, M., Li, J., and Bu, G. (2012) LRP1 in brain vascular smooth muscle cells mediates local clearance of Alzheimer's amyloid-beta. *J. Neurosci.* **32**, 16458-16465
19. Brandenburg, L. O., Konrad, M., Wruck, C. J., Koch, T., Lucius, R., and Pufe, T. (2010) Functional and physical interactions between formyl-peptide-receptors and scavenger receptor MARCO and their involvement in amyloid beta 1-42-induced signal transduction in glial cells. *J. Neurochem.* **113**, 749-760
20. Zhao, Z., Sagare, A. P., Ma, Q., Halliday, M. R., Kong, P., Kisler, K., Winkler, E. A., Ramanathan, A., Kanekiyo, T., Bu, G., Owens, N. C., Rege, S. V., Si, G., Ahuja, A., Zhu, D., Miller, C. A., Schneider, J. A., Maeda, M., Maeda, T., Sugawara, T., Ichida, J. K., and Zlokovic, B. V. (2015) Central role for PICALM in amyloid-beta blood-brain barrier transcytosis and clearance. *Nat. Neurosci.* **18**, 978-987
21. Pflanzner, T., Petsch, B., Andre-Dohmen, B., Muller-Schiffmann, A., Tschickardt, S., Weggen, S., Stitz, L., Korth, C., and Pietrzik, C. U. (2012) Cellular prion protein participates in amyloid-beta transcytosis across the blood-brain barrier. *J. Cerebral Blood Flow Metab.* **32**, 628-632
22. Husemann, J., Loike, J. D., Kodama, T., and Silverstein, S. C. (2001) Scavenger receptor class B type I (SR-BI) mediates adhesion of neonatal murine microglia to fibrillar beta-amyloid. *J. Neuroimmunol.* **114**, 142-150
23. Snyder, E. M., Nong, Y., Almeida, C. G., Paul, S., Moran, T., Choi, E. Y., Nairn, A. C., Salter, M. W., Lombroso, P. J., Gouras, G. K., and Greengard, P. (2005) Regulation of NMDA receptor trafficking by amyloid-beta. *Nat. Neurosci.* **8**, 1051-1058
24. Parri, H. R., Hernandez, C. M., and Dineley, K. T. (2011) Research update: Alpha7 nicotinic acetylcholine receptor mechanisms in Alzheimer's disease. *Biochem. Pharmacol.* **82**, 931-942
25. Zhao, W. Q., Santini, F., Breese, R., Ross, D., Zhang, X. D., Stone, D. J., Ferrer, M., Townsend, M., Wolfe, A. L., Seager, M. A., Kinney, G. G., Shughrue, P. J., and Ray, W. J. (2010) Inhibition of calcineurin-mediated endocytosis and alpha-amino-3-hydroxy-5-methyl-4-isoxazolepropionic acid (AMPA) receptors prevents amyloid beta oligomer-induced synaptic disruption. *J. Biol. Chem.* **285**, 7619-7632
26. Fu, W., Ruangkittisakul, A., MacTavish, D., Shi, J. Y., Ballanyi, K., and Jhamandas, J. H. (2012) Amyloid beta (Abeta) peptide directly activates amylin-3 receptor subtype by triggering multiple intracellular signaling pathways. *J. Biol. Chem.* **287**, 18820-18830
27. Garai, K., Verghese, P. B., Baban, B., Holtzman, D. M., and Frieden, C. (2014) The binding of apolipoprotein E to oligomers and fibrils of amyloid-beta alters the kinetics of amyloid aggregation. *Biochemistry* **53**, 6323-6331
28. Deane, R., Sagare, A., Hamm, K., Parisi, M., Lane, S., Finn, M. B., Holtzman, D. M., and Zlokovic, B. V. (2008) apoE isoform-specific disruption of amyloid beta peptide clearance from mouse brain. *J. Clin. Invest.* **118**, 4002-4013

29. Wang, D., Govindaiah, G., Liu, R., De Arcangelis, V., Cox, C. L., and Xiang, Y. K. (2010) Binding of amyloid beta peptide to beta2 adrenergic receptor induces PKA-dependent AMPA receptor hyperactivity. *FASEB J.* **24**, 3511-3521
30. Killick, R., Ribe, E. M., Al-Shawi, R., Malik, B., Hooper, C., Fernandes, C., Dobson, R., Nolan, P. M., Lourdusamy, A., Furney, S., Lin, K., Breen, G., Wroe, R., To, A. W., Leroy, K., Causevic, M., Usardi, A., Robinson, M., Noble, W., Williamson, R., Lunnon, K., Kellie, S., Reynolds, C. H., Bazenet, C., Hodges, A., Brion, J. P., Stephenson, J., Simons, J. P., and Lovestone, S. (2014) Clusterin regulates beta-amyloid toxicity via Dickkopf-1-driven induction of the wnt-PCP-JNK pathway. *Mol. Psychiatry* **19**, 88-98
31. Vargas, L. M., Leal, N., Estrada, L. D., Gonzalez, A., Serrano, F., Araya, K., Gysling, K., Inestrosa, N. C., Pasquale, E. B., and Alvarez, A. R. (2014) EphA4 activation of c-Abl mediates synaptic loss and LTP blockade caused by amyloid-beta oligomers. *PLoS ONE* **9**, e92309
32. Cisse, M., Halabisky, B., Harris, J., Devidze, N., Dubal, D. B., Sun, B., Orr, A., Lotz, G., Kim, D. H., Hamto, P., Ho, K., Yu, G. Q., and Mucke, L. (2011) Reversing EphB2 depletion rescues cognitive functions in Alzheimer model. *Nature* **469**, 47-52
33. Lacor, P. N., Buniel, M. C., Furlow, P. W., Clemente, A. S., Velasco, P. T., Wood, M., Viola, K. L., and Klein, W. L. (2007) Abeta oligomer-induced aberrations in synapse composition, shape, and density provide a molecular basis for loss of connectivity in Alzheimer's disease. *J. Neurosci.* **27**, 796-807
34. Kam, T. I., Song, S., Gwon, Y., Park, H., Yan, J. J., Im, I., Choi, J. W., Choi, T. Y., Kim, J., Song, D. K., Takai, T., Kim, Y. C., Kim, K. S., Choi, S. Y., Choi, S., Klein, W. L., Yuan, J., and Jung, Y. K. (2013) FcgammaRIIb mediates amyloid-beta neurotoxicity and memory impairment in Alzheimer's disease. *J. Clin. Invest.* **123**, 2791-2802
35. Magdesian, M. H., Carvalho, M. M., Mendes, F. A., Saraiva, L. M., Juliano, M. A., Juliano, L., Garcia-Abreu, J., and Ferreira, S. T. (2008) Amyloid-beta binds to the extracellular cysteine-rich domain of Frizzled and inhibits Wnt/beta-catenin signaling. *J. Biol. Chem.* **283**, 9359-9368
36. Zhao, W. Q., De Felice, F. G., Fernandez, S., Chen, H., Lambert, M. P., Quon, M. J., Krafft, G. A., and Klein, W. L. (2008) Amyloid beta oligomers induce impairment of neuronal insulin receptors. *FASEB J.* **22**, 246-260
37. De Felice, F. G., Vieira, M. N., Bomfim, T. R., Decker, H., Velasco, P. T., Lambert, M. P., Viola, K. L., Zhao, W. Q., Ferreira, S. T., and Klein, W. L. (2009) Protection of synapses against Alzheimer's-linked toxins: insulin signaling prevents the pathogenic binding of Abeta oligomers. *Proc. Natl. Acad. Sci. USA* **106**, 1971-1976
38. Kim, T., Vidal, G. S., Djurisic, M., William, C. M., Birnbaum, M. E., Garcia, K. C., Hyman, B. T., and Shatz, C. J. (2013) Human LilrB2 is a beta-amyloid receptor and its murine homolog PirB regulates synaptic plasticity in an Alzheimer's model. *Science* **341**, 1399-1404
39. Ohnishi, T., Yanazawa, M., Sasahara, T., Kitamura, Y., Hiroaki, H., Fukazawa, Y., Kii, I., Nishiyama, T., Kakita, A., Takeda, H., Takeuchi, A., Arai, Y., Ito, A., Komura, H., Hirao, H., Satomura, K., Inoue, M., Muramatsu, S., Matsui, K., Tada, M., Sato, M., Saijo, E., Shigemitsu, Y., Sakai, S., Umetsu, Y., Goda, N., Takino, N., Takahashi, H., Hagiwara, M., Sawasaki, T., Iwasaki, G., Nakamura, Y., Nabeshima, Y., Teplow, D. B., and Hoshi, M. (2015) Na, K-ATPase alpha3 is a death target of Alzheimer patient amyloid-beta assembly. *Proc. Natl. Acad. Sci. USA* **112**, E4465-4474
40. Dinamarca, M. C., Weinstein, D., Monasterio, O., and Inestrosa, N. C. (2011) The synaptic protein neuroligin-1 interacts with the amyloid beta-peptide. Is there a role in Alzheimer's disease? *Biochemistry* **50**, 8127-8137
41. De Felice, F. G., Velasco, P. T., Lambert, M. P., Viola, K., Fernandez, S. J., Ferreira, S. T., and Klein, W. L. (2007) Abeta oligomers induce neuronal oxidative stress through an N-methyl-D-aspartate receptor-dependent mechanism that is blocked by the Alzheimer drug memantine. *J. Biol. Chem.* **282**, 11590-11601
42. Shankar, G. M., Li, S., Mehta, T. H., Garcia-Munoz, A., Shepardson, N. E., Smith, I., Brett, F. M., Farrell, M. A., Rowan, M. J., Lemere, C. A., Regan, C. M., Walsh, D. M., Sabatini, B.

L., and Selkoe, D. J. (2008) Amyloid-beta protein dimers isolated directly from Alzheimer's brains impair synaptic plasticity and memory. *Nat. Med.* **14**, 837-842
43. Hashimoto, Y., Kaneko, Y., Tsukamoto, E., Frankowski, H., Kouyama, K., Kita, Y., Niikura, T., Aiso, S., Bredesen, D. E., Matsuoka, M., and Nishimoto, I. (2004) Molecular characterization of neurohybrid cell death induced by Alzheimer's amyloid-beta peptides via p75NTR/PLAIDD. *J. Neurochem.* **90**, 549-558
44. Perini, G., Della-Bianca, V., Politi, V., Della Valle, G., Dal-Pra, I., Rossi, F., and Armato, U. (2002) Role of p75 neurotrophin receptor in the neurotoxicity by beta-amyloid peptides and synergistic effect of inflammatory cytokines. *J. Experimental Med.* **195**, 907-918
45. Nimmrich, V., Grimm, C., Draguhn, A., Barghorn, S., Lehmann, A., Schoemaker, H., Hillen, H., Gross, G., Ebert, U., and Bruehl, C. (2008) Amyloid beta oligomers (A beta(1-42) globulomer) suppress spontaneous synaptic activity by inhibition of P/Q-type calcium currents. *J. Neurosci.* **28**, 788-797
46. Lauren, J., Gimbel, D. A., Nygaard, H. B., Gilbert, J. W., and Strittmatter, S. M. (2009) Cellular prion protein mediates impairment of synaptic plasticity by amyloid-beta oligomers. *Nature* **457**, 1128-1132
47. Rushworth, J. V., Griffiths, H. H., Watt, N. T., and Hooper, N. M. (2013) Prion protein-mediated toxicity of amyloid-beta oligomers requires lipid rafts and the transmembrane LRP1. *J. Biol. Chem.* **288**, 8935-8951
48. Rushworth, J. V., and Hooper, N. M. (2010) Lipid Rafts: Linking Alzheimer's Amyloid-beta Production, Aggregation, and Toxicity at Neuronal Membranes. *Int. J. Alzheimers Dis.* **2011**, 603052
49. Um, J. W., Kaufman, A. C., Kostylev, M., Heiss, J. K., Stagi, M., Takahashi, H., Kerrisk, M. E., Vortmeyer, A., Wisniewski, T., Koleske, A. J., Gunther, E. C., Nygaard, H. B., and Strittmatter, S. M. (2013) Metabotropic glutamate receptor 5 is a coreceptor for Alzheimer abeta oligomer bound to cellular prion protein. *Neuron* **79**, 887-902
50. Um, J. W., Nygaard, H. B., Heiss, J. K., Kostylev, M. A., Stagi, M., Vortmeyer, A., Wisniewski, T., Gunther, E. C., and Strittmatter, S. M. (2012) Alzheimer amyloid-beta oligomer bound to postsynaptic prion protein activates Fyn to impair neurons. *Nat. Neurosci.* **15**, 1227-1235
51. Walsh, D. M., and Selkoe, D. J. (2007) A beta oligomers - a decade of discovery. *J. Neurochem.* **101**, 1172-1184
52. Mackic, J. B., Stins, M., McComb, J. G., Calero, M., Ghiso, J., Kim, K. S., Yan, S. D., Stern, D., Schmidt, A. M., Frangione, B., and Zlokovic, B. V. (1998) Human blood-brain barrier receptors for Alzheimer's amyloid-beta 1- 40. Asymmetrical binding, endocytosis, and transcytosis at the apical side of brain microvascular endothelial cell monolayer. *J. Clin. Invest.* **102**, 734-743
53. Origlia, N., Bonadonna, C., Rosellini, A., Leznik, E., Arancio, O., Yan, S. S., and Domenici, L. (2010) Microglial receptor for advanced glycation end product-dependent signal pathway drives beta-amyloid-induced synaptic depression and long-term depression impairment in entorhinal cortex. *J. Neurosci.* **30**, 11414-11425
54. Moore, K. J., El Khoury, J., Medeiros, L. A., Terada, K., Geula, C., Luster, A. D., and Freeman, M. W. (2002) A CD36-initiated signaling cascade mediates inflammatory effects of beta-amyloid. *J. Biol. Chem.* **277**, 47373-47379
55. Stewart, C. R., Stuart, L. M., Wilkinson, K., van Gils, J. M., Deng, J., Halle, A., Rayner, K. J., Boyer, L., Zhong, R., Frazier, W. A., Lacy-Hulbert, A., El Khoury, J., Golenbock, D. T., and Moore, K. J. (2010) CD36 ligands promote sterile inflammation through assembly of a Toll-like receptor 4 and 6 heterodimer. *Nat. Immunol.* **11**, 155-161
56. Izzo, N. J., Staniszewski, A., To, L., Fa, M., Teich, A. F., Saeed, F., Wostein, H., Walko, T., 3rd, Vaswani, A., Wardius, M., Syed, Z., Ravenscroft, J., Mozzoni, K., Silky, C., Rehak, C., Yurko, R., Finn, P., Look, G., Rishton, G., Safferstein, H., Miller, M., Johanson, C., Stopa, E., Windisch, M., Hutter-Paier, B., Shamloo, M., Arancio, O., LeVine, H., 3rd, and Catalano, S. M. (2014) Alzheimer's therapeutics targeting amyloid beta 1-42 oligomers I: Abeta 42 oligomer binding to specific neuronal receptors is displaced by drug candidates that improve cognitive deficits. *PLoS ONE* **9**, e111898

57. Izzo, N. J., Xu, J., Zeng, C., Kirk, M. J., Mozzoni, K., Silky, C., Rehak, C., Yurko, R., Look, G., Rishton, G., Safferstein, H., Cruchaga, C., Goate, A., Cahill, M. A., Arancio, O., Mach, R. H., Craven, R., Head, E., LeVine, H., 3rd, Spires-Jones, T. L., and Catalano, S. M. (2014) Alzheimer's therapeutics targeting amyloid beta 1-42 oligomers II: Sigma-2/PGRMC1 receptors mediate Abeta 42 oligomer binding and synaptotoxicity. *PLoS ONE* **9**, e111899
58. Liu, S., Liu, Y., Hao, W., Wolf, L., Kiliaan, A. J., Penke, B., Rube, C. E., Walter, J., Heneka, M. T., Hartmann, T., Menger, M. D., and Fassbender, K. (2012) TLR2 is a primary receptor for Alzheimer's amyloid beta peptide to trigger neuroinflammatory activation. *J. Immunol.* **188**, 1098-1107
